# 2025 Brazilian guidelines for the management of neuromyelitis optica spectrum disorder in adults and children

**DOI:** 10.1055/s-0045-1812471

**Published:** 2025-11-10

**Authors:** Samira Luisa dos Apóstolos-Pereira, Alfredo Damasceno, Ana Claudia Piccolo, Maria Fernanda Mendes, Maria Lúcia Brito, Regina Alvarenga, Bruna Klein da Costa, Douglas Kazutochi Sato, Doralina Guimarães Brum, Elisa Mello, Felipe von Glehn, Giordani Passos, Guilherme Diogo, Herval Soares Neto, Lis Campos, Manuela Fragomeni, Milena de Sales Pitombeira, Rafael Paterno, Raquel Vassão, Renata Barbosa Paolilo, Tarso Adoni, Thiago Fukuda, Vanessa Daccach, Jefferson Becker, Claudia Cristina Ferreira Vasconcelos

**Affiliations:** 1 Universidade de São Paulo, Faculdade de Medicina, Hospital das Clínicas, Serviço de Neurologia, São Paulo SP, Brazil.; 2 Universidade Estadual de Campinas, Campinas SP, Brazil.; 3 Universidade Municipal de São Caetano do Sul, São Paulo SP, Brazil.; 4 Santa Casa de São Paulo, Faculdade de Ciências Médicas, Disciplina de Neurologia, São Paulo SP, Brazil.; 5 Hospital da Restauração, Recife PE, Brazil.; 6 Universidade Federal do Estado do Rio de Janeiro, Pós-Graduação Stricto Sensu em Neurologia, Rio de Janeiro RJ, Brazil.; 7 Pontifícia Universidade Católica do Rio Grande do Sul, Porto Alegre RS, Brazil.; 8 Santa Casa de Porto Alegre, Hospital da Criança Santo Antônio, Porto Alegre RS, Brazil.; 9 Hospital de Clínicas de Porto Alegre, Serviço de Neurologia, Porto Alegre RS, Brazil.; 10 Universidade Federal do Rio Grande do Sul, Faculdade de Medicina, Departamento de Medicina Interna, Porto Alegre RS, Brazil.; 11 Universidade Estadual Paulista, Faculdade de Medicina de Botucatu, Botucatu SP, Brazil.; 12 Santa Casa de São Paulo, Faculdade de Ciências Médicas, São Paulo SP, Brazil.; 13 Universidade de Brasília, Brasília DF, Brazil.; 14 Pontifícia Universidade Católica do Rio Grande do Sul, Hospital São Lucas, Porto Alegre RS, Brazil.; 15 Hospital Mãe de Deus, Porto Alegre RS, Brazil.; 16 Universidade de São Paulo, Faculdade de Medicina, Hospital das Clínicas, São Paulo SP, Brazil.; 17 Hospital do Servidor Público Estadual de São Paulo, São Paulo SP, Brazil.; 18 Hospital Israelita Albert Einstein, São Paulo SP, Brazil.; 19 Universidade Federal de Sergipe, Aracajú SE, Brazil.; 20 Universidade Tiradentes, Aracajú SE, Brazil.; 21 Hospital da Criança de Brasília, Brasília DF, Brazil.; 22 Hospital Geral de Fortaleza, Divisão de Neurologia, Fortaleza CE, Brazil.; 23 Irmandade da Santa Casa de São Paulo, Serviço de Neurologia, São Paulo SP, Brazil.; 24 Santa Casa de Belo Horizonte, Clínica Neurológica, Belo Horizonte MG, Brazil.; 25 Universidade de São Paulo, Faculdade de Medicina, Hospital das Clínicas, Instituto da Criança, São Paulo SP, Brazil.; 26 Universidade Federal da Bahia, Hospital Universitário Professor Edgar Santos, Salvador BA, Brazil.; 27 Universidade de São Paulo, Faculdade de Medicina de Ribeirão Preto, Hospital das Clínicas, Ribeirão Preto SP, Brazil.

**Keywords:** Neuromyelitis Optica, Aquaporin 4, Disease Management, Therapeutics, Guideline, Consensus

## Abstract

**Background:**

Neuromyelitis optica spectrum disorder (NMOSD) is a debilitating recurrent inflammatory disease of the central nervous system. Establishing management guidelines is essential to optimize patient care in Brazil.

**Objective:**

To combine the Delphi and Grading of Recommendations Assessment, Development, and Evaluation (GRADE) approaches to validate evidence-based guideline statements for NMOSD treatment.

**Methods:**

These guidelines focused on providing recommendations for different scenarios: general management and diagnosis of NMOSD; acute and preventive treatments (including systemic immunosuppressants and anti-B cell, anti-interleukin-6, and anti-complement monoclonal antibodies); and therapeutic approaches in special groups (such as the pediatric population and pregnant women). A literature review based on the Population, Intervention, Comparator, and Outcome (PICO) framework was performed, and studies were evaluated using the GRADE system. An initial questionnaire containing 19 statements was sent to 44 specialists from all regions of Brazil.

**Results:**

Three voting rounds were necessary to reach consensus in all statements. After the first voting round, 17 statements reached consensus (level of agreement > 70%). Two statements failed to reach consensus and were, thus, revised. One of them was segmented into three different statements. After the second voting round, three out of the four revised statements reached consensus. Upon further revision, the last statement was again submitted to voting, reaching consensus in this third round. Overall, agreement was achieved on the 21 proposed statements.

**Conclusion:**

The primary objective in the management of NMOSD is to mitigate the severity of the attacks and prevent relapses, thereby minimizing the risk of irreversible neurological deficits. The statements in the current guideline offer evidence-based recommendations for such management within the Brazilian context.

## INTRODUCTION

Neuromyelitis optica spectrum disorder (NMOSD) is a chronic, relapsing, and debilitating autoimmune inflammatory disease of the central nervous system. It is characterized by severe attacks of optic neuritis, longitudinally-extensive transverse myelitis, and demyelinating syndromes affecting the brain, diencephalon, and brainstem. 1 The disorder predominantly affects non-Caucasian female populations (female:male ratio = 9:1), and the average age of onset is around the fourth decade of life. 2 3

After the discovery of aquaporin-4-immunoglobulin G (AQP4-IgG), a key astrocytic water channel antibody, NMOSD was distinguished from multiple sclerosis (MS). 4 In 2015, the International Panel for Neuromyelitis Optica (NMO) Diagnosis 1 (IPND) recommended AQP4-IgG detection as a fundamental diagnostic biomarker for NMOSD, since it is detected in approximately 75% of NMOSD patients. 5 For those seronegative for AQP4-Ig, approximately 30 to 40% test positive for myelin oligodendrocyte glycoprotein-IgG (MOG-IgG) antibodies. 6 Around 30% of clinically-diagnosed NMOSD patients remain seronegative for both AQP4- and MOG-IgG, the so-called *double-seronegative NMOSD* (DS-NMOSD). 7 When characteristic clinical and neuroimaging findings are present and differential diagnoses are excluded, DS-NMOSD can be diagnosed using the 2015 IPND criteria. 1 Magnetic resonance imaging (MRI) assessment of the brain, optic nerve, and spinal cord is essential to confirm NMOSD-specific imaging and exclude alternative diagnoses such as MS and myelin oligodendrocyte glycoprotein antibody-associated disease (MOGAD). 1

Historically associated with high morbidity and mortality, NMOSD outcomes have improved due to early diagnosis and treatment, reducing mortality to approximately 10% in 10 years. 8 9 Early intervention with acute attack management and immunotherapy has significantly reduced morbidity and improved long-term outcomes. 10 However, irreversible disability remains a major concern due to severe attacks, leading to extensive testing, immediate treatment with high-dose steroids, plasmapheresis, and the frequent need for admission to the Intensive Care Unit. 11 Several recent randomized controlled trials 12 13 14 15 (RCTs) have demonstrated the efficacy of novel monoclonal antibodies (mAbs) such as satralizumab, eculizumab, inebilizumab, and ravulizumab in the treatment of AQP4-IgG-positive NMOSD patients, leading to approvals by regulatory agencies worldwide. However, there are no direct clinical comparisons to establish the best order of treatment administration. 16 Moreover, these drugs are currently not approved for patients living with DS-NMOSD.

Given the disease's complexity and variability, establishing consensus management guidelines is essential to optimize patient care in Brazil and globally. 17 The Delphi method gathers expert opinions to establish guidelines, particularly for rare diseases lacking standardized treatments. 16 The Grading of Recommendations, Assessment, Development, and Evaluation (GRADE) method evaluates the quality of evidence to support treatment decisions. 18 We combined the Delphi 16 and GRADE 18 approaches to prioritize clinical questions and validate evidence-based guideline statements, thus ensuring best-practice recommendations for NMOSD treatment.

## METHODS

A conventional Delphi methodological approach 19 was employed to develop the current guideline. The qualitative study was conducted from March 2023 to October 2024 by the Brazilian Academy of Neurology (Academia Brasileira de Neurologia, ABN, in Portuguese).

**Figure 1:**
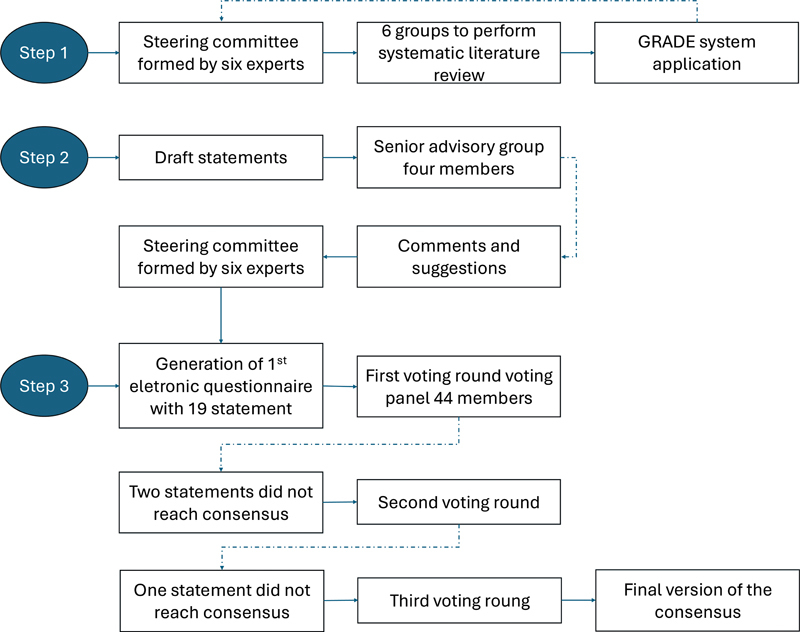
Abbreviations: GRADE, Grading of Recommendations, Assessment, Development, and Evaluation; NMOSD, neuromyelitis optica spectrum disorder. Algorithm of the Delphi method steps used to prepare the 2025 Brazilian NMOSD Consensus.

A steering committee was formed, composed of six experts (ACP, AD, CCFV, JB, MFM, and SLAP) with experience in the diagnosis and treatment of NMOSD ( Figure 1 ). The present guideline focused on providing recommendations for different scenarios: general management and diagnosis of NMOSD; acute and preventive treatments, including systemic immunosuppressive therapy (IST), and anti-B cell, anti-interleukin-6 (anti-IL-6), and anti-complement mAbs; and therapeutic approaches in special groups (such as the pediatric population and pregnant women).

A rapid systematic literature review (RSLR) was conducted to develop statements related to each aspect. To this end, six groups were formed, each consisting of a steering committee member and three specialists with expertise in NMOSD 20 ( Table 1 , Figure 1 ). A structured search in the PubMed, Embase, LILACS, and Google Scholar databases was performed to recover observational studies and double-blinded RCTs written in English, Spanish, and Portuguese, and published from 1999 to 2024. The search strategy was based on the Patient, Intervention, Comparator, Outcome (PICO) framework, as described elsewhere, 21 and developed using the strategy described in Table 2 . All studies considered for full reading were evaluated for the quality of evidence using the GRADE system, and classified accordingly into four levels: high, moderate, low, and very low. 18

**Table 1:** Eligibility criteria and credentials of the expert panel members

1. ≥ 10 years of spelcialty in neurology. 2. One of the following conditions: a. Participation in reference services in a teaching hospital specialized in pleople living with neuromyelitis optica spectrum disorder (NMOSD; pwNMOSD); b. Leading national cohort sudies to investigare NMOSD; and c. Experience in caring for at least 50 pwNMOSD in the last year.

**Table 2:** PICO strategy for the 2025 Brazilian consensus on the treatment of neuromyelitis optica spectrum disorder (NMOSD) in the adult and pediatric populations

Acronym	Description
P - study population	All people living with NMOSD (pwNMOSD), according to diagnostic criteria published since 1999, ^9^ including: ● Adult patients (aged ≥ 18 years); ● Pediatric patients (aged ≤ 17 years); ● Pregnant women with NMOSD, regardless of the aquaporin-4-immunoglobulin G (AQP4-IgG) serostatus.
I - intervention	● Intervention for diagnosis, using the descriptor *AQP4-IgG antibody* ; ● Intervention in the acute phase, using the descriptors *oral and/or intravenous corticosteroids* , *immunoglobulin* , and *therapeutic apheresis* ; and ● Prophylactic intervention to prevent new attacks, using the following descriptors: *oral corticosteroids* , *azathioprine* , *mycophenolate mofetil* , *methotrexate* , *cyclophosphamide* , *rituximab* , *tocilizumab* , *satralizumab* , *inebilizumab* , *eculizumab* , or *ravulizumab* .
C - comparator	● Placebo; ● Corticosteroids; ● Systemic immunosuppressants; and ● Rituximab.
O - Outcome	● Efficacy outcomes: evaluation of the time until the first relapse, reduction in the annualized relapse rate, and disability progression assessed by the Expanded Disability Status Scale; ● Safety and tolerability; and ● Outcomes related to the route and frequency of treatment administration.

### Generation of statements

The statements have four main characteristics: anonymity, interaction, controlled feedback, and statistical group response. They were developed based on the quality classification of the evidence and sent for critical review to a senior advisory group consisting of four neurologists with extensive clinical experience and scientific knowledge about NMOSD. Aspects such as risk of bias or limitations, publication bias, imprecision, inconsistency, and indirect evidence were analyzed in each selected study for the final quality classification of the evidence (online Supplementary Material). The comments and suggestions from the senior group were sent back to the steering committee, and an initial questionnaire containing 19 statements was organized in an electronic form (Google Forms, Alphabet Inc.), with 5 response options categorized by the Likert scale ( *strongly disagree* , *disagree* , *neither agree nor disagree* , *agree* , and *strongly agree* ). Expert opinions were added at the end of the analysis, after the voting rounds, considering the assessment of peculiarities according to the regions of Brazil.

### Voting rounds

An online questionnaire ( **Supplementary Material** , available at https://www.arquivosdeneuropsiquiatria.org/wp-content/uploads/2025/09/ANP-2025.0217-Supplementary-Material.docx ) was sent to 44 main specialists from all regions of Brazil, selected based on their requirements ( Table 1 ) to be part of the voting expert panel and to assist in the development of the final consensus. The steering committee members and the senior specialists did not participate in the voting rounds.

To achieve maximum agreement on each assertion, a maximum of four voting rounds was stipulated; participation in the first two rounds was also a requirement. Each response submission was extracted from Google Forms and transferred to Excel (Microsoft Corp.) spreadsheets, with anonymization concerning the voting specialists, and qualitative and quantitative analyses were conducted. For each voting round, the statements that did not reach a predefined consensus threshold (> 70% of agreement) were revised and reformulated by the steering committee. The revised statements were sent back to the same voting panel, including feedback reports from the qualitative and quantitative analyses. The algorithm depicted in Figure 1 summarizes the steps of the Delphi methodology followed. Standard protocol approvals, registrations, and patient consent were not required for this study. Additional data can be assessed in the Supplementary Material.

## RESULTS

### Overview

The targeted literature search yielded 21 articles on acute management of NMOSD attacks (out of 54 articles identified), 31 articles on oral immunosuppressants (out of 107 articles identified), 34 articles on B-cell depleting therapies (out of 565 articles identified), 9 articles on IL-6 receptor blockade (out of 25 articles identified), 8 articles on complement component 5 (C5) inhibitors (out of 149 articles identified), and 27 articles (out of 147 articles identified) on special situations (pediatric NMOSD, pregnancy, and breastfeeding). Following the GRADE system, all articles included were evaluated (online Supplementary Material). Based on this evidence, 19 draft statements were developed.

### Voting rounds

Three out of the four previously-stipulated voting rounds were necessary to reach consensus in all statements. All voting members (n = 44) participated in all 3 rounds and voted in all statements. After the first voting round, 17 out of the 19 statements reached consensus (level of agreement > 70%). Two statements failed to reach consensus (levels of agreement of 65.9% and 63.6%). Therefore, these statements were revised, and one of them was segmented into three different statements. They were then submitted to a second voting round, upon which three out of the four revised statements reached consensus. The statement that did not reach consensus (level of agreement of 59.1%) was further revised and resubmitted to voting, reaching consensus in this third round (level of agreement of 70.5%).

### Overall results

There was agreement on 21 consensus statements overall. These are discussed in more detail in the sections that follow, which are divided according to the topics of the targeted literature review.

### *General approach to patients with NMOSD diagnosis ( Table 3 )*

**Figure 2:**
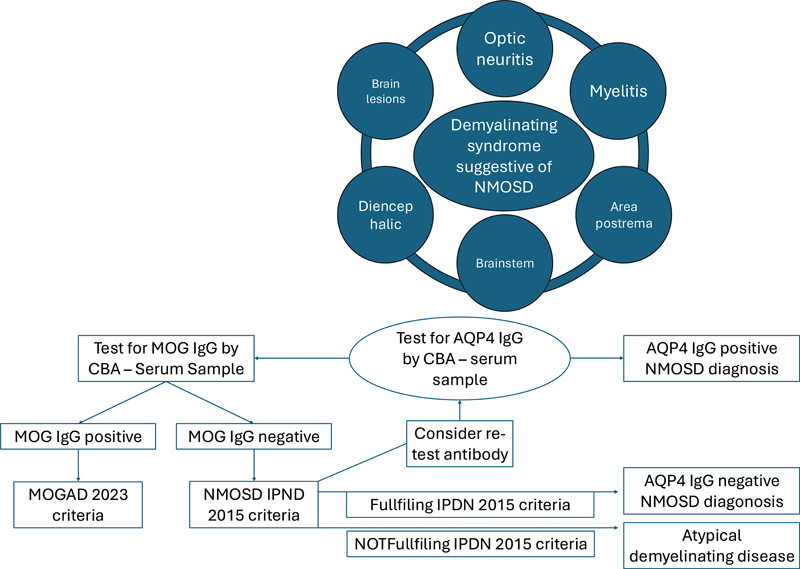
Abbreviations: AQP4-IgG, aquaporin-4-immunoglobulin G; CBA, cell-based assay; MOG-IgG, myelin oligodendrocyte glycoprotein-immunoglobulin G; NMOSD, neuromyelitis optica spectrumdisorder; MOGAD, MOG-IgG-associated disease. Diagnosis of NMOSD based on current NMOSD and MOGAD guidelines.

**Figure 3:**
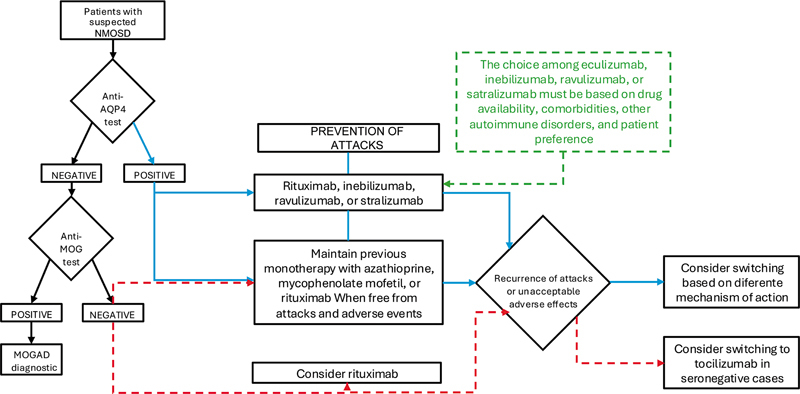
Abbreviations: Anti-AQP4, aquaporin-4-immunoglobulin G; anti-MOG, myelin oligodentrocyte glycoprotein antibody; NMOSD, neuromyelitis optica spectrum disorder; IVMP, intravenous methylprednisolone, IVIG, intravenous immunoglobulin; MOGAD, MOG-IgG myelin oligodendrocyte glycoprotein-immunoglobulin G-associated disease. Diagnosis and treatment of NMOSD based on the AQP4-IgG serostatus, according to the 2025 Brazilian consensus. Treatment recommendations are depicted in the figure. Eculizumab is not included, since its use is not currently approved by the Brazilian Health Regulatory Agency (Agência Nacional de Vigilância Sanitária, ANVISA, in Portuguese) for NMOSD treatment in Brazil. The blue lines indicate the consensus for preventive treatment recommendations for AQP4-IgG-positive patients. The red dashed lines indicate the consensus for AQP4-IgG-negative patients. Considerations regarding the selection among available monoclonal antibodies for preventive treatment are presented in the green dotted box.

Statement 1 reached consensus in round 1 of voting (level of agreement of 95.4%), recommending that patients with clinical presentation suggestive of NMOSD must be tested for AQP4-IgG and, when negative, tested for MOG-IgG. Real-world data 22 provide strong evidence that serum AQP4-IgG predicts NMOSD relapses after an initial attack. The 2015 IPND criteria improved NMOSD diagnosis by 40%, reclassifying 60% of cases and reducing the median time to diagnosis by 1 to 18 months. 1 23 The detection of AQP4-IgG should be performed using appropriate methodologies, most notably cell-based assays (CBAs) employing either fixed or live cells, in serum samples ideally obtained prior to the initiation of immunotherapy. 1 Live CBAs have consistently demonstrated 1 superior diagnostic performance compared to fixed-cell techniques. However, their implementation frequently relies on in-house protocols that can vary across laboratories, posing challenges for standardization and reproducibility—issues that are particularly pronounced in low-income countries, where technical resources and expertise may be limited. 24 Consequently, despite their lower sensitivity, fixed CBAs have been more broadly adopted in the routine clinical practice due to their greater availability and feasibility. 24 Despite optimal methods and timing of testing, approximately 30% of the patients with clinical presentation consistent with NMOSD lack AQP4-IgG (seronegative NMOSD). 25 In a significant proportion of these cases, MOG-IgG is detected. 6 Therefore, it is strongly recommended that all patients under investigation for NMOSD who tested negative for AQP4-IgG should be investigated for MOG antibodies in serum samples using CBA to ensure accurate diagnosis and appropriate management 26 Accurate reporting of MOG-IgG testing requires specifying both the antibody titer and the assay matrix. Titer levels provide valuable quantitative insights that can correlate with disease activity and prognosis, with higher titers more suggestive of pathogenic relevance. Early serum sampling is fundamental for a reliable MOG-IgG diagnosis, as antibody levels may decline over time. 25 While serum testing is generally preferred for its higher sensitivity, cerebrospinal fluid (CSF) analysis may offer additional diagnostic value in select cases, particularly in scenarios of encephalitis and possibly myelitis. Conversely, the usefulness of CSF testing in optic neuritis remains uncertain. Combining detailed titration data with information on assay methodology and sample source enhances diagnostic precision and clinical interpretation. 25 26 ( Figure 2 ). Early antibody detection is essential for accurate diagnosis and the implementation of appropriate therapeutic strategies, 26 mainly in patients exhibiting optic neuritis and myelitis, since NMSOD and MOGAD have different immunological mechanisms and require different therapeutic approaches ( Figure 3 ). Despite advanced detection methods, including live CBA and flow cytometry analysis, approximately 25% of the patients clinically diagnosed with NMOSD remain seronegative for both AQP4 and MOG autoantibodies (DS-NMOSD). 7

**Table 3:** Brazilian Delphi consensus – statements on the general management and diagnosis of NMOSD

Consensus statement	Level of agreement
**General approach to a patient with a NMOSD diagnosis**	
1. We recommend that all patients under investigation for NMOSD be tested for AQP4-IgG antibodies. If negative, then MOG-IgG antibodies should be tested.	95.4%
2. We suggest that the treatment of pwNMOSD should be conducted within a specialized reference center.	79.5%
3. We recommend that: A. Vaccination status should be updated prior to the initiation of preventive therapies in all pwNMOSD; and B. Ongoing monitoring through clinical evaluation and laboratory tests (when indicated) should be performed in NMOSD patients receiving preventive therapy to assess treatment response, adverse events, and manage therapeutic adjustments when necessary.	100%

**Abbreviations:** AQP4-IgG, aquaporin-4-immunoglobulin G; MOG-IgG, myelin oligodentrocyte glycoprotein-immunoglobulin G; NMOSD, neuromyelitis optica spectrum disorder; pwNMOSD, people living with neuromyelitis optica spectrum disorder.

Statement 2 reached consensus (level of agreement of 79.5%) in round 1 of voting, recommending that patients diagnosed with NMOSD receive treatment within specialized reference centers dedicated to this condition. Qualitative real-world data 27 demonstrate that the journey of all people living with NMOSD (pwNMOSD) is challenging due to diagnostic errors and misinformation. Although only qualitative evidence is available, the panel postulated that receiving care in specialized centers may increase the accuracy of diagnosis and the timeliness of appropriate treatment for pwNMOSD, given the complexities associated with the differential diagnosis and the accurate application of diagnostic criteria in scenarios involving diverse populations. 23 24 The strategy of treatment in pwNMOSD requires assessment of infection risk and up-to-date vaccination status, as well as of challenges related to selection, accessibility, and management of preventive therapies (such as serious adverse events). 28 29 Infusion reactions and severe adverse events may occur in patients receiving intravenous MAb therapies, such as rituximab, inebilizumab, eculizumab, ravulizumab, and tocilizumab. It is recommended that these agents be administered in specialized neuroimmunology infusion centers staffed by trained personnel capable of managing infusion-related reactions and monitoring for therapy-specific adverse effects. Implementation of premedication protocols and continuous monitoring during and after infusion are advised to mitigate reaction risk. Additionally, comprehensive preventive strategies should be instituted prior to treatment initiation and maintained throughout therapy. The panel suggested that vigilant clinical monitoring of infections (such as respiratory and urinary infections) and routine laboratory evaluation, including assessment of liver enzymes, complete blood count (including neutrophil and platelet counts), and immunoglobulin levels, are essential to detect cytopenia and hypogammaglobulinemia early, thereby optimizing patient safety and treatment outcomes. 24 27 28 29 In specific clinical scenarios, such as concomitant infections or unexpected relapses, adjustments to the dosing strategy of B-cell-depleting therapies may be required. Rituximab, one of the most widely used and cost-effective immunobiological agents for NMOSD, can be administered at fixed intervals (such as every 6 months) or tailored according to lymphocyte subpopulation counts. Notably, early or unexpected relapses in pwNMOSD receiving rituximab have been associated with paradoxical increases in B-cell–activating factor (BAFF) or premature B-cell repopulation. Monitoring peripheral B-cell counts—particularly those of CD19+ cells—has been shown to predict relapse risk, underscoring the importance of close specialist supervision to guide timely retreatment and optimize dosing schedules. Moreover, genetic variations in Fcγ receptors, particularly *FCGR3A* polymorphisms, have been reported to influence the clinical response to rituximab, suggesting a role of host immunogenetic factors in treatment outcomes. 30 Specifically, lipid (such as cholesterol and triglyceride) levels influenced by the IL-6 blockade must be evaluated, and fibrinogen levels, when deemed necessary. 31

Statement 3 reached consensus (level of agreement of 100% in round 1 of voting), recommending that vaccination status should be up to date before the initiation of preventive therapies. Before starting on a lifelong preventive treatment, the vaccination status must be reviewed and the patient should be screened for infections, including those by the hepatitis B virus (HBV) and tuberculosis. Vaccines are generally safe for pwNMOSD, including those for coronavirus disease 2019 (COVID-19). 32 For patients receiving B-cell-depleting therapies, it is recommended to vaccinate before the initiation of these therapies, particularly for HBV, pneumococcal, influenza, and recombinant adjuvanted zoster vaccines. 32 Live vaccines are contraindicated during IST. 33 In addition, monitoring treatment response and adverse events through clinical evaluation and laboratory exams is mandatory in pwNMOSD under preventive therapy.

### *Acute management of NMOSD attacks*

The panel discussed the acute management of NMOSD attacks ( Table 4 ) based on 3 approaches: 1 g/day of intravenous methylprednisolone (IVMP), therapeutic plasma exchange (TPE), and intravenous immunoglobulin (IVIG). Exacerbations in NMOSD often cause severe disability; thus, prompt, intensive treatment is crucial to suppress relapses and limit CNS damage for better recovery. 34 35 36

**Table 4:** Brazilian Delphi consensus – statements on the acute management of NMOSD attacks

Consensus statement	Level of agreement
**Acute management of NMOSD attacks**	
4. We recommend administering high-dose intravenous methylprednisolone (IVMP; 1g/day for ≥ 5 days) as early as possible for the treatment of NMOSD attacks, regardless of the AQP4-IgG serostatus, to improve outcomes related to attack severity.	97.7%
5. We recommend initiating therapeutic plasma exchange (TPE; for ≥ 5 cycles) as early as possible for the treatment of NMOSD attacks, either alone or associated with high-dose IVMP, regardless of the AQP4-IgG serostatus, to reduce functional disability and increase the likelihood of recovery.	86.4%
6. We do not recommend the use of intravenous immunoglobulin (IVIG) for the treatment of NMOSD attacks, regardless of the AQP4-IgG serostatus, except in patients seropositive for MOG-IgG antibodies.	75%

Abbreviations: AQP4-IgG, aquaporin-4-immunoglobulin G; MOG-IgG, myelin oligodentrocyte glycoprotein-immunoglobulin G; NMOSD, neuromyelitis optica spectrum disorder.

Statement 4 reached consensus (97.7% of agreement in round 1 of voting), recommending that NMOSD attacks must be treated with high doses of IVMP as early as possible. regarding the acute-phase treatment, eight studies were identified: two prospective 36 37 and six retrospective studies. 34 35 38 39 40 41 The main therapeutic strategies evaluated included high-dose IVMP, either alone or in combination with TPE, as well as IVIG administered alone or following IVMP in steroid-refractory cases. The panel members concurred that IVMP is the current standard to treat acute NMOSD attacks and is strongly recommended despite the low grade of evidence. There is a paucity of high-quality studies, and only one study 41 investigated the effectiveness of IVMP as a monotherapy in the acute phase. This recommendation is based on its widespread availability, decades of clinical experience, reduced need for monitoring, and potential to accelerate recovery, particularly when administered early at a dose of 1 g for 3 to 7 consecutive days. 34 41 In the clinical practice, after completing corticosteroid pulse therapy, it is common to maintain 1 mg/kg/day of oral corticosteroids, followed by a gradual, individualized taper. This may vary according to the mechanism of action of the selected preventive therapy. Given the heterogeneity in the clinical approaches, definitive recommendations cannot yet be established. Recovery after an attack in pwNMOSD treated with steroids depends on factors such as early treatment, age, prior immunosuppressant use, elevated CSF protein, and MRI with gadolinium enhancement. 42 Although IVMP is standard for relapses, full recovery is uncommon, and many attacks show limited or no response to therapy. 34 41

Statement 5 reached consensus (86.4% of agreement in round 1 of voting), recommending that NMOSD attacks must be treated with TPE (for ≥ 5 cycles) associated or not with high doses of IVMP and as early as possible (regardless of the AQP4-IgG serostatus). Therapeutic plasma exchange for NMOSD attacks is supported by low-to-moderate quality of evidence under the GRADE framework, but has a strong clinical rationale and observed benefits, leading to a practical recommendation favoring its use. It can be performed concurrently with IVMP or early, as the first-line therapy in severe cases ( Table 5 ). When the response to pulse therapy is incomplete or absent, TPE is recommended for use on alternate days for 5 to 7 cycles, preferably within 5 days of the onset of NMOSD relapse. 34 35 36 37 A retrospective study 34 reviewed 83 cases of NMOSD attacks on patients treated with IVMP alone versus IVMP plus TPE; A reduction in disability (according to the Expanded Disability Status Scale, EDSS) at follow-up occurred in 65% of the combined treatment group, but only in 35% of the group receiving IVMP alone. Two large retrospective cohort studies 38 41 on TPE efficacy in functional recovery concluded that early intervention yields better outcomes, with fewer than 5% of pwNMOSD presenting improvement when TPE was delayed for 3 weeks after the attack. 38 A retrospective cohort 41 of 207 attacks in 105 pwNMOSD identified early TPE initiation and its use as first-line therapy as strong predictors of complete remission. Although TPE appears to be an effective approach to treat steroid-resistant NMOSD attacks and an early intervention improves outcomes, the optimal timing of initiation remains a critical, unresolved issue. 39 43 The use of TPE without steroids was evaluated in a prospective observational study of 30 pwNMOSD, 37 showing functional improvement in 73.3% of the patients and a significant correlation between recovery and early TPE initiation. The AQP4-IgG status did not affect the outcomes. Current evidence supports a “the sooner, the better” approach for both high-dose IVMP and TPE. However, access to tertiary centers offering TPE remains a major barrier, especially in low-income regions. In practice, delayed healthcare access can hinder timely treatment, potentially worsening prognosis. Of note, immunoadsorption, a distinct therapeutic apheresis technique, is limited to ultraspecialized centers and was not considered in the present consensus.

**Table 5:** Severe relapse criteria in pwNMOSD 43 115

Severe relapses in pwNMOSD include: 1. Optic neuritis: bilateral optic neuritis or severe monocular visual loss (visual acuity worse than 20/200); and 2. Acute myelitis: clinical presentation consistent with transverse/complete myelitis, major motor deficits (muscle strength lower than II out of V on the MRC scale), or bladder dysfunction.

Abbreviations: MRC, Medical Research Council Scale for muscle strength; pwNMOSD, people living with neuromyelitis optica spectrum disorder.

Statement 6 reached consensus (75% of agreement in round 1 of voting) on the assumption that IVIG is not indicated for acute exacerbations of NMOSD. This recommendation aligns with the absence of high-quality evidence per the GRADE to support IVIG use. Alone or in combination, IVIG has not shown sufficient benefit in treating NMOSD relapses, regardless of the AQP4-IgG status. The data available consistently demonstrate that IVIG is less effective than steroids or TPE. 44 In steroid-resistant attacks, IVIG is inferior to TPE in reducing pathogenic antibody levels and improving disability outcomes. 45 Based on current evidence, the panel agreed that the most effective strategy for severe NMOSD relapses remains the combined use of high-dose corticosteroids and plasmapheresis.

### *NMOSD preventive treatment*

The panel addressed preventive treatment across 18 scenarios: 6 for AQP4-IgG-positive NMOSD, 5 concerning DS-NMOSD patients meeting the 2015 IPND criteria, 1 for patients stable on long-term immunosuppression, 3 for pediatric cases, and 3 for women during pregnancy or breastfeeding. The preventive treatments were categorized by AQP4-IgG status, as robust evidence 1 supports NMOSD AQP4-IgG-positive as a distinct disease entity ( Figure 3 ), and treatment recommendations are now supported by high-level evidence under the GRADE framework ( Table 6 ). Patients fulfilling clinical and neuroimaging criteria for NMOSD but testing negative for AQP4-IgG should be re-evaluated for differential diagnoses, including MS and MOGAD ( Figures 2 3 ). Our recommendations for AQP4-IgG-negative NMOSD apply only to those who do not meet the latest MS criteria and are also negative for MOG-IgG. Treatment in this subgroup must be individualized due to diagnostic uncertainty, absence of reliable biomarkers for diagnosis, and a scarce literature on treatment ( Tables 7 8 ). Recommendations for special populations, irrespective of the AQP4-IgG status—such as pediatric patients and pregnant or breastfeeding women—are presented in Tables 9 10 . These guidelines do not apply to MOG-IgG-positive individuals who are classified under MOGAD, a distinct clinical entity.

**Table 6:** Brazilian Delphi consensus – Statements on the preventive management of NMOSD attacks in AQP4-IgG-positive patients

Consensus statement	Level of agreement
**Prevention of NMOSD attacks in AQP4-IgG-seropositive patients**	
7. We recommend rituximab monotherapy, initiated at diagnosis, after the first attack, or following relapse due to the failure of previous treatments, for the prevention of attacks and disability in adults and children with NMOSD, regardless of the AQP4-IgG serostatus.	77.3%
8. We recommend treatment with eculizumab, inebilizumab, ravulizumab, or satralizumab, as monotherapy, a tdiagnosis, after the first attack, or following relapse due to the failure of previous treatments, for the prevention of attacks and disability in adults (aged ≥ 18 years) with AQP4-IgG-positive NMOSD.	95.5%
9. We recommend that the choice among eculizumab, inebilizumab, ravulizumab, or satralizumab consider factors such as drug availability, comorbidities, other autoimmune disorders, and patient preference, since efficacy and safety have not been compared in head-to-head studies in AQP4-IgG-seropositive pwNMOSD.	97.7%
10. We do not recommend the combined therapy with two or more monoclonal antibodies (e.g., eculizumab, inebilizumab, ravulizumab, rituximab, satralizumab, or tocilizumab) in AQP4-IgG-seropositive NMOSD patients.	95.5%
11. We do not recommend azathioprine, cyclophosphamide, oral steroids, methotrexate, or mycophenolate mofetil, as monotherapy, for first-line preventive therapy in adults with AQP4-IgG-positive NMOSD.	75%*

Abbreviations: AQP4-IgG, aquaporin-4-immunoglobulin G; NMOSD, neuromyelitis optica spectrum disorder; pwNMOSD, people living with neuromyelitis optica spectrum disorder.

Note: *Agreement reached in round 2 of voting.

**Table 7:** Brazilian Delphi Consensus – statements on preventive management of NMOSD attacks in AQP4-IgG-negative patients

Consensus statement	Level of agreement
**Prevention of NMOSD attacks in AQP4-IgG-seronegative patients**	
7. We recommend monotherapy with rituximab at diagnosis, after the first attack, or following relapse due to the failure of previous treatments, for the prevention of relapses and disability in adults or children with NMOSD, regardless of the AQP4-IgG serostatus.	77.3%
12. We suggest that azathioprine or mycophenolate mofetil may be used as initial preventive therapy in AQP4-IgG-negative patients (children and adults), only in the initial phase of the disease and when other options are not available. These oral therapies should be used with caution, given the absence of high-quality evidence supporting their application.	84.1%
13. We suggest tocilizumab monotherapy at diagnosis, after the first attack, or following relapse due to the failure of previous treatments, for the prevention of attacks and disability in AQP4-IgG-negative adults with NMOSD.	70.5%*
14. We do not recommend, at this time, treatment with other B-cell-depleting therapies (e.g., ocrelizumab, ofatumumab, or ublituximab) for pwNMOSD, given the lack of supporting evidence.	97.7%

Abbreviations: AQP4-IgG, aquaporin-4-immunoglobulin G; NMOSD, neuromyelitis optica spectrum disorder; pwNMOSD, people living with neuromyelitis optica spectrum disorder.

Note: *Agreement reached in round 3 of voting.

**Table 8:** Brazilian Delphi Consensus – atatements on long-term preventive management of NMOSD attacks in clinically-stable patients

Consensus statement	Level of agreement
**Long-term prevention of NMOSD attacks in clinically-stable patients**	
15. We suggest that in all pwNMOSD (adults or children) who are relapse-free and without adverse events, the use of azathioprine, mycophenolate mofetil, or rituximab as preventive therapy may be maintained, regardless of the AQP4-IgG serostatus.	90.1%

Abbreviations: AQP4-IgG, aquaporin-4-immunoglobulin G; NMOSD, neuromyelitis optica spectrum disorder; pwNMOSD, people living with neuromyelitis optica spectrum disorder.

**Table 9:** Brazilian Delphi consensus – Statements on preventive management of NMOSD attacks in children – special situations

Consensus statement	Level of agreement
**Preventive treatment in children with NMOSD**	
7. We recommend rituximab as monotherapy at diagnosis, after the first attack, or after relapse due to the failure of previous treatments, for the prevention of relapses and disability in adults or children with NMOSD, regardless of the AQP4-IgG serostatus.	77.3%
16. We recommend satralizumab, at diagnosis, after the first attack, or after relapses due to the failure of previous treatments, as monotherapy or combined with oral immunosuppressants, for the prevention of relapses and disability in adolescents aged ≥ 12 years with NMOSD who are AQP4-IgG seropositive.	93.2%
17. We suggest that preventive therapy with azathioprine or mycophenolate mofetil be used with caution in children with NMOSD, regardless of the AQP4-IgG serostatus, and only when rituximab and satralizumab are contraindicated.	86.4%
18. We do not recommend monotherapy with cyclophosphamide, oral steroids, or methotrexate for relapse prevention in children with NMOSD, regardless of the AQP4-IgG serostatus.	81.8%

Abbreviations: AQP4-IgG, aquaporin-4-immunoglobulin G; NMOSD, neuromyelitis optica spectrum disorder.

**Table 10:** Brazilian Delphi consensus – statements on preventive management of NMOSD attacks according to AQP4-IgG status: special situations

Consensus statement	Level of agreement
**Preventive treatment in NMOSD during pregnancy and breastfeeding**	
19. We suggest the use of rituximab during pregnancy and breastfeeding in women with NMOSD, regardless of the AQP4-IgG serostatus, when the patient provides informed consent and is closely monitored in collaboration with the obstetrics team.	84.1%
20. We recommend that treatment with cyclophosphamide, methotrexate, or mycophenolate mofetil be avoided during pregnancy and breastfeeding in women with NMOSD, regardless of the AQP4-IgG serostatus, due to teratogenic and toxicity risks.	100%
21. We do not suggest the use of eculizumab, inebilizumab, ravulizumab, or satralizumab during pregnancy and breastfeeding in women with NMOSD, given the current lack of evidence to support their application in this context.	86.4%

Abbreviations: AQP4-IgG, aquaporin-4-immunoglobulin G; NMOSD, neuromyelitis optica spectrum disorder.

### Preventive treatment in AQP4-IgG-positive NMOSD

Statements 7, 8, 9, 10, and 11 were developed to guide the preventive treatment in AQP4-IgG-positive NMOSD, and they are presented in Table 6 .

Statement 7 reached the consensus (77.3% of agreement in round 1 of voting) that rituximab treatment is recommended at the moment the diagnosis is established, after the first attack, and after relapse due to the failure of previous treatments, for the prevention of attacks and disability in adult and children diagnosed with NMOSD, regardless of the AQP4-IgG serostatus. This recommendation is supported by a placebo-controlled randomized trial, called “A multi-center, randomized, double-blind, placebo-controlled trial to determine the efficacy of rituximab against a relapse of neuromyelitis optica spectrum disorders with anti-aquaporin 4 antibody” (RIN-1), 46 which enrolled 38 adults with AQP4-IgG-positive NMOSD, randomized 1:1 to receive rituximab (375 mg/m ^2^ weekly for 4 weeks, followed by 2 g every 6 months) or placebo. While 37% of the placebo group experienced relapses, none in the rituximab group relapsed. This trial provides high-level evidence, according to the GRADE framework, that rituximab significantly reduces relapse risk in adults with AQP4-IgG-positive NMOSD. Additional evidence of low-to-moderate quality from observational and open-label studies supports its effectiveness across other outcomes and subgroups (online Supplementary Material). For instance, the study called “An open-label extension study following the RIN-1 study to investigate the efficacy of rituximab against a relapse of neuromyelitis optica” (RIN-2), 47 which compared rituximab to the placebo arm of RIN-1, reported an annualized relapse rate (ARR) of 0.035 with rituximab versus 0.321 in the placebo group, with stable EDSS scores throughout the study period. Regarding safety, adverse events of grades ≥ 3 occurred in 4 to 6% of the patients in the RIN-1 and RIN-2 trials. A meta-analysis 48 of 36 studies (n = 1,542) reported infrequent, mostly mild-to-moderate adverse events, with infusion reactions and infections being the most common. These risks can be mitigated through strategies such as premedication, infusion rate adjustments, and vaccination protocols.

Statement 8 reached consensus (95.5% of agreement in round 1 of voting), recommending the treatment with eculizumab, inebilizumab, ravulizumab, or satralizumab, as a monotherapy in AQP4-IgG-seropositive pwNMOSD at the moment the diagnosis is established, after the first attack, and after relapse due to the failure of previous treatments, for the prevention of attacks and disability in adults (aged ≥ 18 years). This statement is supported by RCTs for eculizumab, 14 49 inebilizumab, satralizumab, 15 31 50 and ravulizumab, 13 according to the GRADE framework (evidence of moderate-to-high quality).

The recommendation of eculizumab to prevent relapses in AQP4-IgG-positive NMOSD is based on the phase-3 trial called “A Randomized Controlled Trial of Eculizumab in AQP4 Antibody-positive Participants With NMO” (PREVENT), 14 which enrolled 143 adult. After about 211 weeks, the relapse rate was of 3% in the eculizumab group compared to 43% in the placebo group (hazard ratio [HR] = 0.06; 95%CI = 0.02–0.20; *p* < 0.001). As a secondary endpoint, eculizumab monotherapy was associated with a significantly lower rate of worsening of the disability status according to the EDSS (4.8% versus 38.5% in the placebo group). 49

The recommendation of ravulizumab is supported by the open-label trial with an external comparator called “A Phase 3, External Placebo-Controlled, Open-Label, Multicenter Study to Evaluate the Efficacy and Safety of Ravulizumab in Adult Patients With Neuromyelitis Optica Spectrum Disorder (NMOSD)” (CHAMPION-NMOSD). 13 A total of 58 AQP4-IgG-positive NMOSD adults received IV loading doses of ravulizumab ranging from 2,400 to 3,000 mg, followed by 3,000 to 3,600 mg every 8 weeks. No relapses occurred during the follow-up, although the single-arm design and use of an external comparator are study limitations.

The recommendation of satralizumab (120 mg via subcutaneous [SC] injection every 4 weeks [Q4w]; as monotherapy or in combination with oral ISTs) for relapse prevention in AQP4-IgG-seropositive NMOSD patients was supported by 2 RCTs and one observational study (moderate-quality evidence for ARR reduction and time until first relapse). 15 31 50 The analysis of disability (EDSS) generally were included as secondary endpoints in the studies evaluated.

The recommendation of inebilizumab is supported by a single RCT (high-quality evidence): “A Double-masked, Placebo-controlled Study With Open-label Period to Evaluate the Efficacy and Safety of MEDI-551 in Adult Subjects With Neuromyelitis Optica and Neuromyelitis Optica Spectrum Disorders” 12 (N-Momentum), a phase-2/3 RCT that enrolled 230 patients, 93% of whom were AQP4-IgG-seropositive. The patients received 2 IV doses of inebilizumab (300 mg on days 1 and 15) or placebo. For the primary outcome—time until first relapse—inebilizumab demonstrated a 73% relative risk reduction compared to placebo (12% versus 39%; HR = 0.27; 95%CI = 0.15–0.50; *p* < 0.0001). Disability worsening, measured by the EDSS, was assessed as a secondary endpoint and showed less progression in the inebilizumab group compared to the placebo group. Additional long-term data from the open-label extension of N-MOmentum 51 showed that 77% of the patients remained relapse-free after 4 years of continuous treatment. The limitations include the relatively-short duration of the blinded follow-up (28 weeks) and the underrepresentation of Black or African-American patients, who comprised only 9% of the study population.

Statement 9 reached consensus (97.3% of agreement in round 1 of voting), recommending that the choice among eculizumab, inebilizumab, ravulizumab, or satralizumab should consider factors such as drug availability, comorbidities or other autoimmune disorders, and patient preference, since efficacy and safety have not been compared in head-to-head studies. According to the GRADE framework, there is low-level evidence to support clinical decision-making, and previous publications 52 were heterogeneous in their assessment of efficacy, using different endpoints and relapse definitions. Moreover, real-world evidence 53 54 on the use of novel mAbs in individuals with NMOSD remains scarce, primarily due to significant disparities in therapeutic access across geographic regions and diverse populations. While these therapies are approved and available in many high-income countries, access is still limited or delayed in low- and middle-income countries, representing a major challenge and contributing to substantial global disparities in NMOSD care. 17

From a clinical standpoint, mechanisms of action may assist in the selection of appropriate therapies in patients with comorbidities. Of note, eculizumab, ravulizumab, and inebilizumab have also been also approved for the treatment of generalized myasthenia gravis (gMG), which can co-occur with NMOSD, 55 and, in such cases, they may offer dual therapeutic benefits. 56 Moreover, IL-6 receptor-blocking mAbs (that is, satralizumab and tocilizumab) may be advantageous in patients with NMOSD and coexisting autoimmune diseases—such as rheumatoid arthritis, giant cell arteritis, or systemic sclerosis—in which IL-6 signaling plays a pivotal role in pathogenesis. 15

Safety profiles are a critical consideration. Complement inhibitors (eculizumab and ravulizumab) require meningococcal vaccination before initiation, 14 whereas patients treated with inebilizumab or satralizumab require monitoring for infections and hematological abnormalities. 12 15

Dosing frequency and administration routes significantly impact adherence to treatment and quality of life. Ravulizumab is administered intravenously every 8 weeks, offering a significant advantage over eculizumab, which requires biweekly dosing. 13 Inebilizumab, on the other hand, is administered intravenously every 6 months, a regimen that may be particularly appealing to patients residing in rural or remote areas, since it minimizes the need for frequent travel. 12 In contrast, satralizumab is administered monthly by SC injections, which enables self-injection at home and offers a significant advantage for patients with difficult venous access or those seeking to reduce hospital visits. 57 Finally, regarding adverse events, satralizumab treatment was generally well tolerated, with only a slightly higher risk of infections compared to placebo. 57 Thus, the panel agreed that therapeutic decisions in NMOSD should be individualized, taking into account not only efficacy and safety, but also comorbidities, drug accessibility, dosing convenience, and patient preference.

Statement 10 reached consensus (95.5% of agreement in round 1 of voting), not recommending the use of combined mAb therapy in patients diagnosed with NMOSD. Combining mAb therapy in NMOSD is not recommended due to insufficient evidence on its safety and efficacy. Most clinical trials were designed to evaluate monotherapy. 16 At initial phases, 58 most studies kept pwNMOSD on a stable dose of previous IST, but not mAbs. In this regard, low-quality evidence describes 59 the use of IST associated with mAbs, even though combining these therapies could increase the risk of adverse effects such as infections. Future research may provide new insights into its potential benefits.

Statement 11 reached consensus (75% of agreement in round 2 of voting), recommending against the use of azathioprine, cyclophosphamide, oral steroids, methotrexate, or mycophenolate mofetil as first-line preventive monotherapy in adults with AQP4-IgG-seropositive NMOSD. The original version of this statement included adults and children, but it failed to reach consensus (63% of agreement) due to insufficient evidence. The final recommendation focused solely on adults, acknowledging the lack of pediatric data, which is addressed separately in statement 16.

Due to the lack of high-to-moderate-quality evidence regarding the efficacy and safety of these drugs, in contrast to the high-quality evidence for early use of mAbs such as rituximab, the use of immunosuppressants as monotherapy as first-line treatment in adults with AQP4-IgG-seropositive NMOSD was not recommended. In addition, rituximab is associated with improvements in cost-efficacy 60 and quality of life. 61 The key limitations of traditional immunosuppressants include: the lack of RCTs, with most studies being retrospective, with small sample sizes (especially in comparator studies), inconsistent treatment protocols, and a high risk of bias (example: concomitant corticosteroid use), and inferior efficacy compared to mAbs, as trials allowing background immunosuppressants (such as azathioprine, mycophenolate) showed worse outcomes than those using mAbs. 31

The use of azathioprine is supported by seven observational studies (n = 641) that showed reduced ARR, 8 62 63 64 65 66 67 while confirming its inferiority to rituximab, 64 mycophenolate mofetil, 67 and cyclophosphamide. 66 Regarding disability, the EDSS scores improved by 0.98 points with rituximab versus 0.44 points with azathioprine ( *p* < 0.001). A significantly higher proportion of patients achieved relapse-free status with rituximab (78.8%) compared to azathioprine (54.3%; *p* = 0.033). 64 New relapses were observed in 33.5% of the patients, and the impact on disability was highly variable. A prospective cohort study compared azathioprine, mycophenolate mofetil, and lower doses of rituximab; no difference was found between azathioprine and mycophenolate mofetil, while low doses of rituximab were significantly more effective in reducing relapse rates, with higher relapse-free status and a better tolerability profile. The concomitant use of azathioprine and corticosteroids in some studies 62 63 65 66 may have favored the results observed. There was a significant rate of adverse events (35%) and discontinuation (16%) while using azathioprine. 8 The use of oral prednisolone is supported by two small Japanese retrospective observational studies 68 69 with a small number of cases and classified as very low-quality evidence; both studies reported a significant reduction in ARR (0.49 versus 1.48 and 0.21 versus 0.98; *p* < 0.01) and stabilization of the EDSS scores (change: +0.02 versus +0.89; *p* < 0.01) if compared with no medication. Adverse events, ranging from mild to severe, were reported (online Supplementary Material). The use of methotrexate (after the previous use of other drugs) is supported by limited, very low-quality evidence from two retrospective observational studies 70 71 showing a reduction in ARR (from 1.39 pretreatment to 0.18 posttreatment, *p* < 0.005; and from 0.97 to 0.00, *p* < 0.05), albeit relapses still occurred in 57% of the patients 70 (online Supplementary Material).

The evaluation on the use of mycophenolate mofetil is based on the results of 16 observational studies involving 786 pwNMOSD treated with it as monotherapy. Reduced relapse rates were observed in nine studies; 72 73 74 75 76 77 78 79 80 however, the overall evidence remains insufficient to support a definitive treatment recommendation due to its predominantly low- to very low-quality (online Supplementary Material). In a cohort of 90 AQP4-IgG-positive patients receiving monotherapy, high and moderate doses of micophenolate mofetil led to greater reductions in AAR and EDSS scores. 73 In comparative analyses, 67 73 77 81 82 the efficacy of micophenolate mofetil was lower than that of rituximab. Adding all patients from the 16 studies, new relapses were reported in 23% of the total number of patients treated, and adverse effects, in 18%, ranging from mild to severe dose-dependent. 75

### Preventive treatment for patients with AQP4-IgG-negative NMOSD

Statements 7 and 12 to 14 were developed to guide the preventive treatment in patients with AQP4-IgG-negative NMOSD, and they are shown in Table 7 .

Statement 7 reached the consensus (77.3% of agreement in round 1 of voting) that rituximab treatment is recommended at the moment the diagnosis is established, after the first attack, and after relapse due to the failure of previous treatments, for the prevention of attacks and disability in adults and children diagnosed with NMOSD, regardless of the AQP4-IgG serostatus.

Evidence of preventive treatment in AQP4-IgG-negative NMOSD is scarce, according to the GRADE framework. Even so, there was no difference in relapse rates and disability scores between AQP4-IgG seropositive and seronegative NMOSD in a recent meta-analysis of patients treated with rituximab. Similar findings were reported in retrospective studies 83 84 that included approximately 20% seronegative patients, which showed reductions in ARR and stabilization or improvement in disability scores. Observational evidence also suggests that rituximab has comparable efficacy in AQP4-IgG-negative patients 85 and in pediatric populations, 86 supporting its use across a broader spectrum of NMOSD phenotypes. Moreover, rituximab is associated with a better quality of life in pwNMOSD, and its early use is associated with a better cost-effectiveness profile in an economic model. 60 61 While these findings are based on lower-certainty evidence, they reinforce rituximab's potential usefulness beyond AQP4-IgG-positive adult patients.

Key considerations for seronegative patients include the identification of clinical heterogeneity and the need for the exclusion of MOGAD and other disease mimics. Regarding comparative efficacy, rituximab remains the first-line therapy due to its consistent outcomes across NMOSD subtypes. 85

The panel recommended that, in AQP4-IgG-positive adults with NMOSD, azathioprine, mycophenolate, steroids, methotrexate, or cyclophosphamide should be avoided as monotherapy due to low-quality evidence regarding efficacy and safety. Rituximab was preferred in this population. In AQP4-IgG-negative NMOSD, rituximab was also recommended, despite the lower evidence quality, supported by real-world data.

Statement 12 reached consensus (84.1% of agreement in round 1 of voting), suggesting that azathioprine and mycophenolate mofetil should be used as preventive therapy in all AQP4-IgG-negative pwNMOSD (children and adults) only in the initial phase of the disease and when other drugs (rituximab and tocilizumab) are not possible. These IST should be prescribed with caution, given the absence of high-quality evidence.

Due to limited evidence and the lack of proven efficacy of mAbs in patients with AQP4-IgG–negative NMOSD, therapeutic options remain restricted. However, there is growing support for the use of azathioprine and mycophenolate mofetil in seronegative patients. The panel's suggestion is supported by the significant reduction in relapse rates with azathioprine use, regardless of the AQP4-IgG serostatus. 62 64 Moreover, four observational studies 77 78 79 82 have reported comparable reductions in relapse rates with mycophenolate mofetil among seronegative individuals. Still, the evidence is limited by methodological constraints, and we advise cautious therapeutic decision-making.

Furthermore, three comparative observational studies support the superiority of rituximab 64 82 and tocilizumab 87 over azathioprine. The evidence favoring rituximab is rated as moderate-quality by the GRADE, indicating a reasonable level of confidence. A multicenter, phase-2, open-label trial 87 with 59 patients demonstrated a significant reduction in the risk of relapse with the use of tocilizumab (HR = 0.17; 95%CI = 0.04–0.76), with 81% of the patients remaining relapse-free at 48 weeks, compared to 37% of those treated with azathioprine. This moderate certainty reflects a potential bias from the open-label design and small sample size, but the results suggest that tocilizumab is indeed a promising alternative for the treatment of high-risk NMOSD patients. Of note, adverse events were comparable between the groups, supporting an acceptable safety profile for tocilizumab. 87

Statement 13 reached consensus (70.5% of agreement in round 3 of voting), suggesting the use of tocilizumab as monotherapy—either at the moment the diagnosis is established, after the first attack, or following relapse due to the failure of previous treatments—for the prevention of attacks and disability in adults with AQP4-IgG-negative NMOSD. Four observational studies 88 89 90 91 of very low-quality evidence and one moderate-quality RCT 87 have indicated that tocilizumab (8 mg/kg IV Q4W) may reduce ARR in AQP4-IgG–positive and -negative NMOSD. The treatment was well tolerated, with a lower risk of adverse events (primarily infections and transaminase elevations) compared to azathioprine. The panel recommends diligent monitoring of the treatment (statement 3). Of note, an initial statement was designed, in which we suggested the administration of this treatment to adults (aged ≥ 18 years) with NMOSD, regardless of the AQP4 serostatus. However, due to low-quality evidence regarding the efficacy of tocilizumab when compared with evidence from mAbs evaluated in an RCT for AQP4-IgG-positive patients, this statement did not reach consensus after the first (agreement of 65.9%) or second (agreement of 59.1%) voting rounds. Therefore, this statement was revised and finally reached consensus (agreement of 70.5%) after the third voting round, with tocilizumab treatment being recommended only for AQP4-IgG-negative NMOSD patients.

Statement 14 reached consensus (97.7% of agreement in round 1 of voting), not recommending the use of other B-cell-depleting therapies (ocrelizumab, ofatumumab, or ublituximab) for patients diagnosed with NMOSD, regardless of the AQP4-IgG serostatus, given the current absence of supporting evidence. A study assessing ofatumumab in pwNMOSD was registered, but its completion date has passed, and its status has not been updated in more than 2 years. To date, no studies have evaluated the efficacy of other B-cell-depleting therapies other than rituximab and inebilizumab in pwNMOSD.

### Preventive treatment in patients with NMOSD: special clinical situations

Statement 15 was developed to guide the preventive management in patients with NMOSD who remain clinically stable and relapse-free over prolonged periods while under ongoing IST, regardless of the AQP4-IgG serostatus or age ( Table 8 ). Its significance arises from two considerations: first, stable and relapse-free patients are typically excluded from RCTs; and second, no biomarker is currently available to predict relapse in this population.

Statement 15 reached the consensus (90.1% of agreement in round 1 of voting) that preventive therapy with azathioprine, mycophenolate mofetil, or rituximab can be maintained in all patients (adults or children) already undergoing treatment and who are both relapse-free and present no adverse events, regardless of the AQP4-IgG serostatus. This statement is based on pivotal RCTs assessing mAbs in pwNMOSD, all of which required the inclusion of patients with active disease despite previous treatments. The RIN-1 trial 46 enrolled patients with documented relapses within the preceding 2 years. The PREVENT trial 14 included patients with ≥ 2 relapses in the previous 12 months or ≥ 3 in the previous 24 months, including at least 1 in the last year. The open-label CHAMPION-NMOSD extension 13 enrolled patients who presented at least 1 relapse in the preceding 12 months. In the SAkuraSky study, 57 patients with ≥2 relapses within 24 months (including ≥1 in the last year) were enrolled while maintaining their previous immunosuppressants, whereas in the study called “A Multicenter, Randomized, Addition to Baseline Treatment, Double-Blind, Placebo-Controlled, Phase 3 Study to Evaluate the Efficacy and Safety of Satralizumab (SA237) in Patients With Neuromyelitis Optica (NMO) and NMO Spectrum Disorder (NMOSD)” (SAkuraStar), 57 the patients had experienced ≥ 1 relapse in the previous 12 months, discontinuing any previous immunosuppressants.

Given this context, there is no high-level-evidence, according to the GRADE framework, to guide switching from conventional ISTs to mAbs in patients with stable NMOSD. The panel members agreed that patients who are relapse-free and without adverse events on their current therapy do not need to be switched to a new one. While waiting for further real-world data and the identification of new biomarkers able to predict treatment failure, the panel acknowledged that therapeutic changes in this subgroup may be considered on a case-by-case basis.

### Preventive treatment in children with NMOSD

Statements 7, 16, 17, and 18 were developed to the guide preventive treatment in children with NMOSD ( Table 9 ).

Statement 7 reached consensus (77.3% of agreement in round 1 of voting), recommending rituximab as monotherapy at the moment the diagnosis is established, after the first attack, and after relapse due to the failure of previous treatments, for the prevention of attacks and disability in adults or children diagnosed with NMOSD, regardless of the AQP4-IgG serostatus. According to the GRADE framework, the use of rituximab has a high level of evidence for AQP4-IgG-positive NMOSD patients, and a low to moderate evidence for patients with DS-NMOSD and children. This statement reiterates the recommendation of rituximab for the prevention of relapses and disability in children with NMOSD, regardless of the AQP4 serostatus. This is supported by four retrospective observational studies involving 91 pediatric patients. 92 93 94 95 Three studies 92 93 94 analyzed the ARR, 2 of which observed a decrease in ARR either from 2.50 to 0.14 ( *p* < 0.001), 92 or from 1 to 0, 94 while a third noted a stable ARR of 0.25. 93 Two studies 93 95 evaluated patients who remained relapse-free during treatment, with 87.5% 95 and 31.5% 93 of patients free from relapses over periods ranging from 6 to 150 months. No data were available on the time until the first relapse. Adverse events were only assessed in one study, 92 which reported infusion reactions in three patients and one case of neutropenia. Given the reliance on observational data, caution is advised when using rituximab in pediatric patients, emphasizing the need for monitoring for infusion-related reactions and neutropenia, as per statement 3.

Statement 16 reached consensus (93.2% of agreement in round 1 of voting), recommending satralizumab as monotherapy or combined with oral immunosuppressants as treatment at the moment the diagnosis is established, after the first attack, and after relapse due to the failure of previous treatments, for the prevention of attacks and disability in adolescents (aged ≥ 12 years) with AQP4-IgG-positive NMOSD. This statement is based on a high-quality study 15 that analyzed the efficacy and adverse effects of satralizumab treatment in pwNMOSD. Considering the rarity of NMOSD in children, conducting RCTs with sufficient statistical power exclusively for pediatric patients remains extremely challenging.

Statement 17 reached consensus (86.4% of agreement in round 1 of voting), suggesting that azathioprine and mycophenolate mofetil may be used as preventive therapies in children with NMOSD, regardless of the AQP4-IgG serostatus, only with caution and when rituximab and satralizumab are contraindicated. This recommendation is supported by five retrospective observational studies 92 93 94 95 96 that evaluated the use of azathioprine in pediatric patients, all of which were of low-quality evidence regarding efficacy, according to the GRADE framework. Three studies reported a reduction in the ARR before and after azathioprine treatment, from 0.89 to 0.65 in 11 AQP4-IgG-positive patients, 94 from 1.69 to 0.59 in 39 AQP4-IgG-positive patients, 92 and from 1.26 to 0.40 in 10 patients irrespective of the AQP4-IgG serostatus 96 . Only two studies 93 95 evaluated the proportion of relapse-free patients using azathioprine as monotherapy or in combination with oral corticosteroids: Umeton et al. 93 reported that 33% of the patients were relapse-free, while McKeon et al. 95 reported a rate of 50% of relapse-free patients. There is no available data regarding the time until the first relapse, relapse prevention, or changes in disability accumulation.

The use of mycophenolate is supported by four observational studies, 92 93 94 95 all of very low-quality evidence according to the GRADE framework. Three studies 92 93 94 evaluated the ARR regardless of the AQP4-IgG serostatus, and the mean ARR across the studies was of 0.44. In two studies this decreased after treatment, from 0.98 to 0.28, 94 and from 1.04 to 0.72. 92 Another study 95 reported that 66% of the patients remained relapse-free over a 12- to 24-month period. In none of these studies disability accumulation or its reduction were reported. Although observational studies have shown reductions in the ARR and a low frequency of side effects, high- or moderate-quality evidence is currently lacking to confirm the efficacy of mycophenolate mofetil for relapse reduction, time until relapse, disability accumulation or reduction, or its safety in children and adolescents. Only low-quality studies have evaluated the safety of azathioprine and mycophenolate mofetil in pediatric patients with NMOSD. Among those treated with azathioprine, the reported adverse events include lymphopenia, infections, gastrointestinal disturbances, and elevated liver enzymes. Therefore, caution is recommended if azathioprine or mycophenolate are required to be used in pediatric populations, with particular attention to the increased risk of infections and gastrointestinal complications. Active monitoring for infections, vaccination status, and laboratory monitoring of liver enzymes and cytopenias are strongly advised during treatment with either medication. 97

Statement 18 reached consensus (81.8% of agreement in round 1 of voting), not recommending treatment with cyclophosphamide, oral steroids, or methotrexate as preventive monotherapy for children with NMOSD, regardless of the AQP4-IgG serostatus. The use of methotrexate was reported 95 in 1 patient who had no relapses over 36 months; in the same study, on the other hand, two children relapsed during treatment with cyclophosphamide.

### Preventive treatment in NMOSD during pregnancy and breastfeeding ( Table 10 )

Statement 19 reached consensus (84.1% of agreement in round 1 of voting), suggesting that rituximab may be considered during pregnancy and breastfeeding in women with NMOSD, regardless of the AQP4-IgG serostatus, as long as the patient consents and treatment is closely monitored in collaboration with the obstetrics team. In eight low-quality studies, 98 99 100 101 102 103 104 105 the use of azathioprine, rituximab, and prednisolone during pregnancy and the postpartum period was described. These studies concluded that maintaining the IST throughout pregnancy and postpartum reduces the ARR and prevents disability accumulation. Data on relapse rates, disability progression, or adverse effects on the fetus or pregnant patients were not reported. These treatments should only be administered with the patient's full informed consent and under close monitoring by the neurology and obstetrics teams. Despite the lack of high- or moderate-evidence efficacy studies on the use of azathioprine in patients with NMOSD, its use is supported in statements 12 and 13 in cases in which rituximab is unavailable or in patients already receiving azathioprine with a favorable therapeutic response.

Teratogenicity has not been identified in humans and, based on safety data from pregnant patients with inflammatory bowel disease and organ transplant recipients, azathioprine has recently been recognized 106 as a safe immunosuppressant during pregnancy. Breastfeeding is compatible with azathioprine use, as drug transfer into breast milk is minimal. 107 Nevertheless, treatment should be accompanied by informed patient consent and close monitoring throughout the perinatal period.

Statement 20 reached consensus (100% of agreement in round 1 of voting), contraindicating treatment with cyclophosphamide, methotrexate, or mycophenolate mofetil during pregnancy and breastfeeding in women with NMOSD, regardless of the AQP4-IgG serostatus, due to teratogenic and toxicity risks. In safety studies in women of childbearing age and pregnant women with NMOSD, methotrexate was contraindicated due to teratogenic and abortifacient effects, 108 cyclophosphamide, due to known embryofetal toxicity, 109 and mycophenolate mofetil, due to the risk of spontaneous abortion and congenital anomalies. 110 Cyclophosphamide was also contraindicated during breastfeeding because it is excreted in breast milk at toxic levels. 111

Statement 21 reached consensus (86.3% of agreement in round 1 of voting), not recommending treatment with eculizumab, inebilizumab, ravulizumab, or satralizumab during pregnancy and breastfeeding in women with NMOSD, given the absence of evidence to support such an indication. There is no safety data on the use of these medications in pregnant or lactating women. Agencies such as the Brazilian Health Regulatory Agency (Agência Nacional de Vigilância Sanitária, ANVISA, in Portuguese) or the United States Food and Drug Administration (FDA) have not yet classified the risks of these drugs during pregnancy and breastfeeding.

## DISCUSSION

The current consensus represents the first initiative to capture expert perspectives on the management of NMOSD in a continental developing country. Developed using the Delphi methodology, a validated approach for scientific discussions among expert panels, the present guideline aims to generate knowledge on topics with limited scientific evidence, such as NMOSD. A selected group of neuroimmunology experts contributed to the development and validation of the consensus statements on the management of NMOSD, based on the quality of evidence to support therapeutic decision-making directed at people living with this disease.

Additionally, social determinants of health are known to impact outcomes in pwNMOSD. This may be worse in scenarios in which disparities between public and private healthcare systems affect access to timely diagnosis and treatment, such as the access to AQP4-IgG testing, which, in Brazil, is available in the private sector, but not within the public system routinely. In Brazil, the incorporation of new diagnostic tools and treatments is evaluated by the National Commission for the Incorporation of Technologies into the Unified Health System (Comissão Nacional de Incorporação de Tecnologias no Sistema Único de Saúde, CONITEC, in Portuguese), which advises the Ministry of Health on the technologies and clinical guidelines that should be included into the Brazilian Unified Health System (Sistema Único de Saúde, SUS, in Portuguese). Currently, there are no therapeutic options for NMOSD available through either the SUS or private health insurance. This limitation derives from the absence of a defined protocol within SUS and the lack of regulatory definitions from the Brazilian National Health Agency (Agência Nacional de Saúde, ANS, in Portuguese), which prevents private-sector physicians from prescribing appropriate medications for NMOSD. The panel concurs that the development of an official national guideline for NMOSD management should be prioritized to ensure tailored care for patients in a country with a high prevalence of the disease. 112

Given the complexity and distinct pathophysiology of immune-mediated neurological diseases, the present study specifically focused on NMOSD seropositive for AQP4-IgG and NMOSD seronegative for both AQP4-IgG and MOG-IgG, in agreement with the 2015 IPND diagnostic criteria. 1 It is noteworthy to emphasize that the current consensus does not address, and its recommendations are not intended for, other immune-mediated conditions such as MOGAD, once this is now recognized as distinct entity. Neither do our recommendations encompass high-risk syndromes suggestive of NMOSD, not fulfilling 2015 IPND diagnostic criteria, 1 which are seronegative for both AQP4-IgG and MOG-IgG, including isolated or recurrent optic neuritis, chronic relapsing idiopathic optic neuritis, isolated or recurrent transverse myelitis, and brainstem syndromes. Brazilian guidelines underscore the urgent need for early and widespread access to AQP4-IgG testing. In early 2025, CONITEC recommended its inclusion in the SUS for patients with clinical and radiological features of NMOSD. Despite regulatory approval, the test remains largely inaccessible in most public healthcare settings. This is expected to improve early diagnosis and pave the way for the formulation of treatment planning strategies in this country with continental dimensions.

The primary objective in the management of patients diagnosed with NMOSD is to mitigate the severity of the attacks and prevent relapses, thereby minimizing the risk of irreversible neurological deficits from recurrent episodes. Regarding acute-stage management, it was reemphasized that exacerbations in pwNMOSD result in severe residual disability. Therefore, the treatment of relapses must begin promptly and aggressively, which is crucial to prevent severe disability. The goals of the acute-phase therapy include suppressing relapses, minimizing CNS damage, and promoting optimal functional recovery. The evidence supports the principle of ‘the sooner, the better’ for both high-dose methylprednisolone and apheresis. From a pragmatic perspective, in certain regions of Brazil, significant delays in accessing tertiary care centers where plasmapheresis may be available remain a challenge. Nonetheless, such delays should not preclude the administration of optimal treatment strategies during the acute phase. This emphasizes the critical importance of ensuring that these patients are adequately monitored and managed in specialized centers.

The accessibility and affordability of recently-approved treatments for NMOSD are likely to vary across countries and regions, significantly influencing clinical decisions regarding the timing of treatment initiation and the choice of the therapeutic agent. 16 17 58 Recently, several prospective RCTs have led to the FDA approval of the first three immunotherapies for patients with AQP4-IgG-positive NMOSD: eculizumab in June 2019, inebilizumab in June 2020, and satralizumab in August 2020. In addition, rituximab was approved for NMOSD in Japan in June 2022 based on the results of an investigator-initiated phase-II/III clinical study; and in May 2023, the European Medicines Agency (EMA) approved ravulizumab for the treatment of AQP4-IgG-positive NMOSD. In Brazil, ravulizumab, satralizumab, and inebilizumab were respectively approved by ANVISA in 2022, 2020, 2023 respectively. 24 To date, there are no head-to-head trials or direct clinical evidence to guide the best treatment strategy for NMOSD, and the current recommendations are based on expert opinions and consensus. 16 17 58 113 114 In upper-middle-income countries such as Brazil, the heterogeneous healthcare landscape—including limited access to serum diagnostics, acute-phase interventions like therapeutic apheresis, and high-efficacy monoclonal antibody therapies—compounds the challenges of clinical management and underscores the urgent unmet needs that this consensus aims to address.

In conclusion, these guidelines, developed through an evidence-based modified Delphi process, constitute a significant step towards the standardization of care. The statements herein proposed aim to address a critical gap by providing evidence-based recommendations for the management of patients with NMOSD within the Brazilian context.
